# Supplementary material for: Genomic and Phenotypic Characterization of a Drug-Susceptible Acinetobacter baumannii Reveals Increased Virulence-Linked Traits and Stress Tolerance
Source: Biology (Basel). 2025 Sep 5;14(9):1201. doi: 10.3390/biology14091201 (PMC12467113; doi:10.3390/biology14091201)
Supplement: Supplementary file 1 [file biology-14-01201-s001.zip › Supplementary_Material.pdf]

Figure S1. Bacterial growth on (A) Columbia blood agar supplemented with 5% defibrinated horse blood; (B) Skim milk agar. No observable hemolytic or proteolytic activities was detected in either the ATCC19606 and HKAB-1 strains.

(A)

ATCC19606 HKAB-1

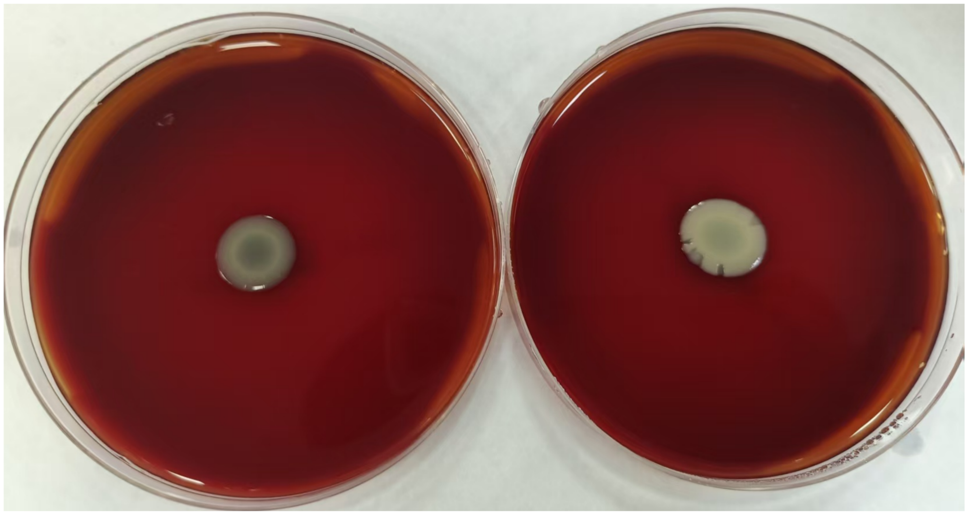

(B)

ATCC19606 HKAB-1

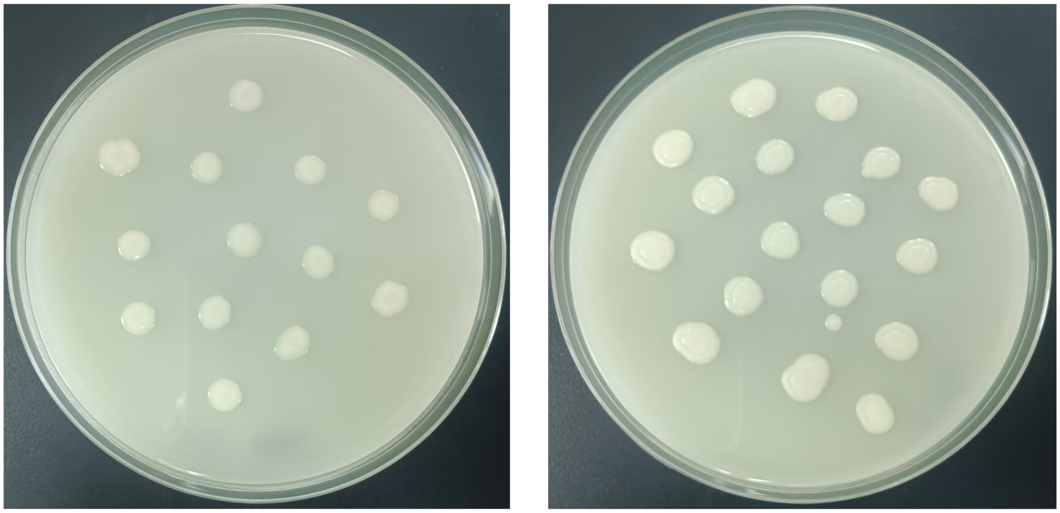

Figure S2. Comparison of COG category gene counts between *A. baumannii* ATCC19606 and HKAB-1. Bar plots display gene counts across five functional categories: C (energy production and conversion), E (amino acid transport and metabolism), P (inorganic ion transport and metabolism), M (cell wall, membrane, and envelope biogenesis), and G (carbohydrate transport and metabolism). HKAB-1 (green) consistently exhibits higher gene counts than ATCC19606 (blue), indicating potential genomic adaptations that may underlie its enhanced growth kinetics. COG functional classification was performed according to the methodology established by Tatusov et al. 2000 [1] and updated in the latest COG database release by Galperin et al. 2021 [2].

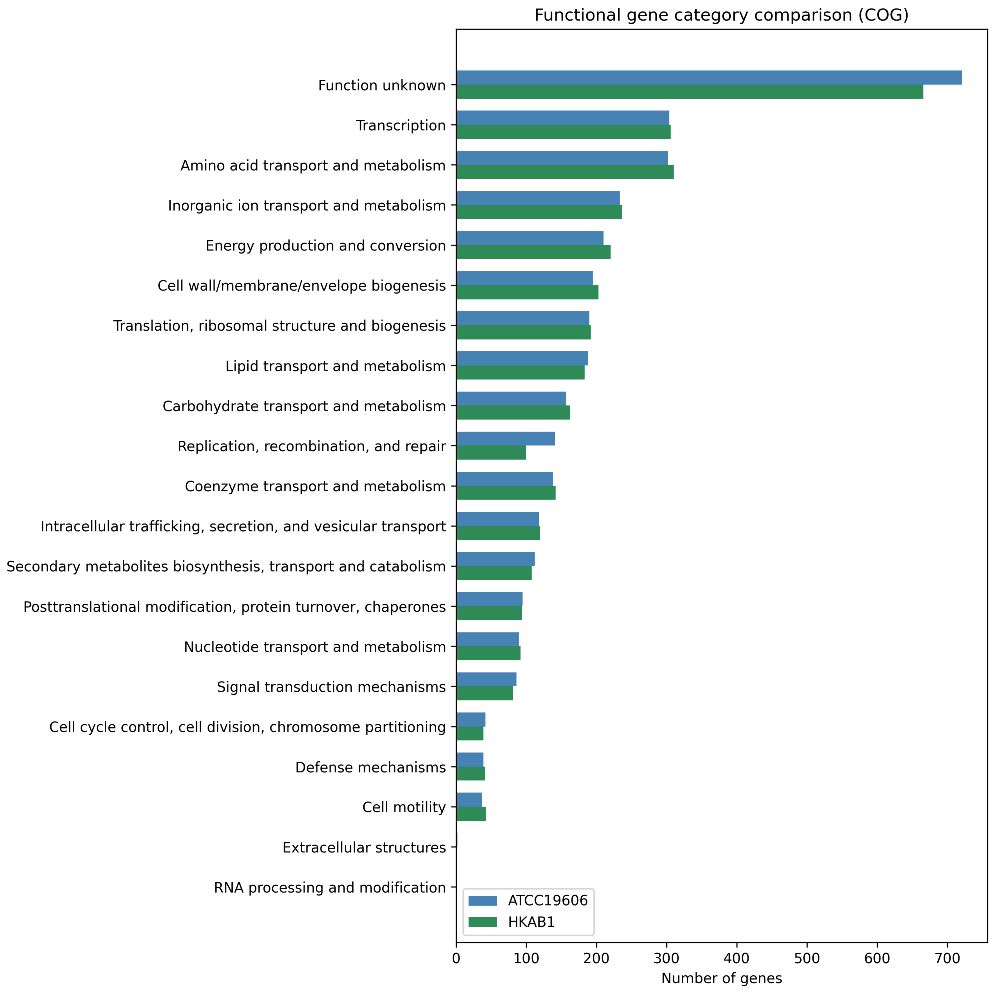

Figure S3. Sequence alignment and phylogeny of *A. baumannii* PilA variants. **(A)** Phylogenetic tree illustrating the evolutionary relationships among PilA protein sequences from representative *A. baumannii* strains, e.g. BIDMC57 (WP_031953428), ATCC19606 (QNT85320), BJAB0715 (AGQ08183), NIPH-601 (ENW54968), NIPH-615 (ENU71103), AB5075 (AKA30090), AB307-0294 (ATY42799), 135867 (KCX54016), NIPH-410 (EPG41173), HKAB-1 (XTQ57233), NIPH-329 (ENW47547), Ab44444 (EKB42086), OIFC137 (EJG13706), ATCC17978 (ABO13574), ACICU (ACC58690), 6935 (KCW32129), 121738 (EXG37371), Strain-1 (KHO16593), and NIPH-80 (ENW77893). GenBank accession numbers are provided in parentheses. **(B)** Multiple sequence alignment of PilA proteins. Amino acid residues are denoted by (*) for identity, (:) for strong similarity, and (.) for weak similarity.

(A)

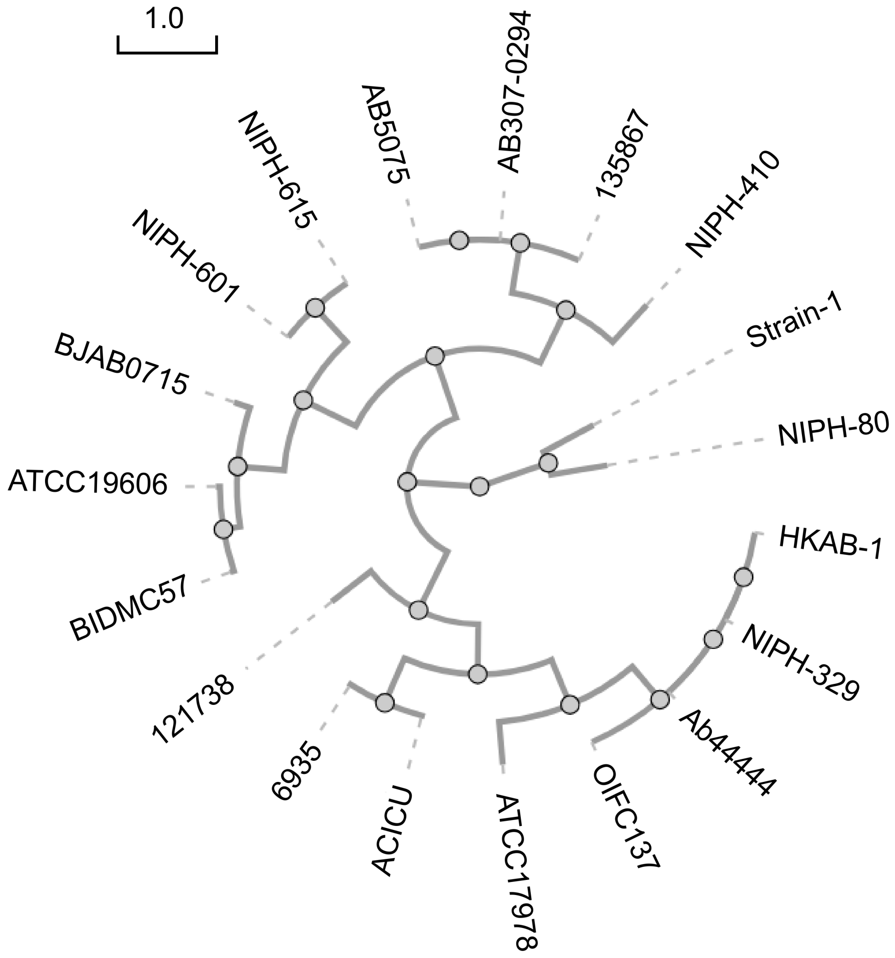

(B)

```
                          10        20        30        40        50        60
BIDMC57         MNAQKGFTLIELMIVVAIIGILAAIAIPAYQNYIAKSQASEAFTLADGLKTTINTNLQAG
ATCC19606       MNAQKGFTLIELMIVVAIIGILAAIAIPAYQNYIAKSQASEAFTLADGLKTTINTNLQAG
BJAB0715        MNAQKGFTLIELMIVVAIIGILAAIAIPAYQSYIAKSQASEAFTLADGLKTTIATNLQAG
NIPH-601        MNAQKGFTLIELMIVVAIIGILAAIAIPAYQDYIARSQMSEALNLSDSLKTNVTETYGQI
NIPH-615        MNAQKGFTLIELMIVVAIIGILAAIAIPAYQDYIARSQMSEALNLSDSLKTNVTETYGQI
AB5075          MNAQKGFTLIELMIVVAIIGILAAIAIPQYQTYIAKSQVSRAVSESGSLKTVIEDCLNN-
AB307-0294      MNAQKGFTLIELMIVVAIIGILAAIAIPQYQTYIAKSQVSRAVSESGSLKTVIEDCLNN-
135867          MNAQKGFTLIELMIVVAIIGILAAIAIPQYQTYIAKSQVSRAVSESGSLKTVIEDCLNN-
NIPH-410        MNAQKGFTLIELMIVVAIIGILAAIAIPQYQNYVGRSNVAAAVQTLTSNKTGLENYVMEN
HKAB-1          MNAQKGFTLIELMIVVAIIGILAAIAIPAYQNYVARSQATAGYSEISNMRTGYDTELNDG
NIPH-329        MNAQKGFTLIELMIVVAIIGILAAIAIPAYQNYVARSQATAGYSEISNMRTGYDTELNDG
Ab44444         MNAQKGFTLIELMIVVAIIGILAAIAIPAYQNYVARSQATAGYSEISNMRTGYDTELNDG
OIFC137         MNAQKGFTLIELMIVVAIIGILAAIAIPAYQNYVARSQATAGYSEISNMRTGYDTELNDG
ATCC17978       MNAQKGFTLIELMIVVAIIGILAAIAIPQYQKYTARSQITAALAEISPGKTQFELALSEG
ACICU           MNAQKGFTLIELMIVVAIIGILAAIAIPAYQNYIAKSQVSTGLADITAGKTNAETKLAEG
6935            MNAQKGFTLIELMIVVAIIGILAAIAIPAYQNYIAKSQVSTGLADITAGKTNAETKLAEG
121738          MNAQKGFTLIELMIVVAIIGILAAIAIPAYQNYIAKSQVNRVYGELSSLKTAAEQLILDG
Strain-1        MNAQKGFTLIELMIVVAIIGILAAVALPAYQDYTVRAKVSEAILAGSACRTTVTEVYQSA
NIPH-80         MNAQKGFTLIELMIVVAIIGILAAIAIPAYRSYIATSYGSQAKGGLDAVIGKVQACIQTG
                ************************:*:* *: *   :

                          70        80        90       100       110       120
BIDMC57         TCFAGGATAVTAADKVSGKYGDAEIGGSAPN----CTITYTFKSSGVSNKLTSTKIVMNV
ATCC19606       TCFAGGATAATAADQVAGKYGDAEIGGTAPN----CTITYTFKSSGVSTKLTSKQIVMNV
BJAB0715        TCFANGSAAATATDSVTGKYGKAEILADTTSGSNGCGIKYTFNASNVSAKLKNGVIGIAV
NIPH-601        GTFTGISSGANGIPASTTVVGKYVSGVAVQDG----VITATMASSNVSKGIQGKTLTLTP
NIPH-615        GTFTGISSGANGIPASTTVVGKYVSGVAVQDG----VITATMASSNVSKGIQGKTLTLTP
AB5075          GKTTVGEAAGECAIGATGSNILDGAAQSGETLAAGTGVPQVTLANTGAATIVATFGNSAS
AB307-0294      GKTTVGEAAGECAIGATGSNILDGAAQSGETLAAGTGVPQVTLANTGAATIVATFGNSAS
135867          GKTTVGEAAGECAIGATGSNILDGAAQSGETLAAGTGVPQVTLANTGAATIVATFGNSAS
NIPH-410        GFFPDGKTAEQAEVKTNGVVTTPFIPDQRLKEGIGIVQPSFGTIELQQKSTTAGTGNIVI
HKAB-1          TAITSLSQVGFT--ATSSGACSAIGVTNFGNDGAATNAITCTLKGNPKIAGKIISLSRSA
NIPH-329        TAITSLSQVGFT--ATSSGACSAIGVTNFGNDGAATNAITCTLKGNPKIAGKIISLSRSA
Ab44444         TAITSLSQVGFT--ATSSGACSAIGVTNFGNDGAATNAITCTLKGNPKIAGKIISLSRSA
OIFC137         TAITSLSQVGFT--ATSSGACSAIGVTNFGNDGAATNAITCTLKGNPKIAGKIISLSRSA
ATCC17978       TANSVNNNPAAIGLKSTTNNCSAVNVTANG----TTGTIACTLQGSATITGGVLTLTRSA
ACICU           LTAALTDVEALG----LQKSTNACSTITTSIGTNGASNITCTLKGTSQINSKKIEWIRDA
6935            LTAALTDVTTLG----LQQSTNACAITAN-IGTNGASNITCTLKGTSQINGKKIEWIRDA
121738          KSSASATDLGYN--TSNLLNSTPTVSVNPNNDGTVSIAGTLGTSATSSVNGAVVTLSRTA
Strain-1        SGSLPAANGWGCEVGSGTSAASKYVQQVQTDQNGVITVTTTNDASLKAAGSSTIVMTPTL
NIPH-80         VGCEDLN-------TTKELAAAKYQNRLSVVAPADGQVAEATSATLKWKNEGCIVQVAAA
```

```
                       130       140       150       160
BIDMC57         SETGILTK----------------NSGTDTPVELLPQSFVAS-------
ATCC19606       SETGILTK----------------NSSTNAPAELLPQSFTAS-------
BJAB0715        SENGVLKKST-------------VTATTSTLNEYLPQSFSGS-------
NIPH-601        TDKGGSVT---------------WACTSNAEQKYLPKACTGGTS-----
NIPH-615        TDKGGSVT---------------WACTSNAEQKYLPKACTGGTS-----
AB5075          TALKSTP----TTVTWTRTTDGTWTCESTAAEKYNSSACPAA-------
AB307-0294      TALKSTP----TTVTWTRTTDGTWTCESTAAEKYNSSACPAA-------
135867          TALKSTP----TTVTWTRTTDGTWTCESTAAEKYNSSACPAA-------
NIPH-410        TFNTGNPGIKGNKVQLHRAEDGTWTCETTIDAKYAAKSCSKVSALTAAS
HKAB-1          TGAWTCST------DIATTDEFLPKGCTGGGTPAVGAITTL--------
NIPH-329        TGAWTCST------DIATTDEFLPKGCTGGGTPAVGAITTL--------
Ab44444         TGAWTCST------DIATTDEFLPKGCTGGGTPAVGAITTL--------
OIFC137         TGAWTCST------DIATTDEFLPKGCTGGGTPAVGAITTL--------
ATCC17978       DVAAASGVNA----NVGGWTCSITKGTTDISSVIAPKGCTII-------
ACICU           DNATNGTTG-------------AWRCKTDVAENLRPKSCGAS-------
6935            DNATNGTTG-------------AWRCKTDVAENLRPKSCGAS-------
121738          TGAWSCRVT--------------ASNNGGWKSSFVPSGCAAS-------
Strain-1        ANG-TAMTSTNIGTSVGAWKCGPGAGATGMPTKFLPGSCRGA-------
NIPH-80         ADGGIAYKFNFITGKATAAQCAKGAGLD--AAADLDGALN---------
```

References

1. Tatusov, R.L.; Galperin, M.Y.; Natale, D.A.; Koonin, E.V. The COG database: a tool for genome-scale analysis of protein functions and evolution. *Nucleic Acids Res.* **2000**, *28*, 33–36.
2. Galperin, M.Y.; Wolf, Y.I.; Makarova, K.S.; Vera Alvarez, R.; Landsman, D.; Koonin, E.V. COG database update: focus on microbial diversity, model organisms, and widespread pathogens. *Nucleic Acids Res.* **2021**, *49*, D274–D281.
